# Cost and cost-effectiveness analysis of a community mobilisation intervention to reduce intimate partner violence in Kampala, Uganda

**DOI:** 10.1186/s12889-016-2883-6

**Published:** 2016-02-29

**Authors:** Christine Michaels-Igbokwe, Tanya Abramsky, Karen Devries, Lori Michau, Tina Musuya, Charlotte Watts

**Affiliations:** London School of Hygiene & Tropical Medicine, London, UK

**Keywords:** Cost analysis, Cost effectiveness analysis, Economic evaluation, Intimate partner violence community mobilisation

## Abstract

**Background:**

Intimate partner violence (IPV) poses a major public health concern. To date there are few rigorous economic evaluations of interventions aimed at preventing IPV in low-income settings. This study provides a cost and cost effectiveness analysis of SASA!, a community mobilisation intervention to change social norms and prevent IPV.

**Methods:**

An economic evaluation alongside a cluster randomised controlled trial. Both financial and economic costs were collected retrospectively from the provider’s perspective to generate total and unit cost estimates over four years of intervention programming. Univariate sensitivity analysis is conducted to estimate the impact of uncertainty in cost and outcome measures on results.

**Results:**

The total cost of developing the SASA! Activist Kit is estimated as US$138,598. Total intervention costs over four years are estimated as US$553,252. The annual cost of supporting 351 activists to conduct SASA! activities was approximately US$389 per activist and the average cost per person reached in intervention communities was US$21 over the full course of the intervention, or US$5 annually. The primary trial outcome was past year experience of physical IPV with an estimated 1201 cases averted (90 % CI: 97–2307 cases averted). The estimated cost per case of past year IPV averted was US$460.

**Conclusion:**

This study provides the first economic evaluation of a community mobilisation intervention aimed at preventing IPV. SASA! unit costs compare favourably with gender transformative interventions and support services for survivors of IPV.

**Trial registration:**

ClinicalTrials.gov #NCT00790959

## Background

Intimate partner violence (IPV) is the most common form of violence experienced by women worldwide [1], with 30 % of all women aged 15 years and above having experienced physical and/or sexual violence at some point in their lives [1]. Across sub-Saharan Africa, 30–65 % of women over the age of 15 have experienced violence at the hands of an intimate partner [1]. In Uganda specifically, more than half of women who have ever been married have experienced physical and/or sexual violence perpetrated by a spouse or partner and 33 % have experienced physical and/or sexual violence in the past 12 months [2].

In addition to being a violation of human rights, exposure to violence is strongly associated with negative physical, sexual and mental health outcomes [3, 4]. IPV also places an economic burden on society with the global combined cost of physical IPV, sexual violence against women and female homicide committed by intimate partners estimated at nearly US $4.8 trillion (adjusted to 2013 US) [5].

In low income settings a variety of interventions aimed at preventing IPV have been implemented and yet few have been rigorously evaluated to determine their effectiveness and cost-effectiveness. This lack of data makes it difficult for decision makers to assess value for money and prioritise interventions in this field [6, 7]. Thus there is a clear need for more evidence informed programming.

This study presents a cost and cost effectiveness analysis of SASA!, a community mobilisation intervention aimed at reducing intimate partner violence in Kampala, Uganda. The intervention was evaluated using a cluster randomised controlled trial. Intervention costs, alongside adjusted risk differences for past year experience of IPV among partnered women in intervention sites versus control sites, are used to generate a cost effectiveness ratio with the cost per case of past year physical IPV averted as the primary outcome.

## Methods

### The SASA! intervention

SASA! - An Activist Kit for Preventing Violence against Women and HIV [8] is an intervention designed by Raising Voices and implemented by Center for Domestic Violence Prevention (CEDOVIP), two non-governmental organisations based in Kampala, Uganda. The overall aim of the intervention is to prevent violence against women and reduce HIV/AIDS risk. SASA! recognises that IPV and HIV risk occurs in a community context and is influenced by attitudes, norms and power imbalances between men and women. A community mobilisation approach is used to stimulate change at individual, relationship, community and societal levels using a four stage process, represented by the acronym SASA! [9, 10].

1. Start: Start thinking about violence against women and HIV/AIDS as interconnected issues and foster power within staff and community members to address these issues.
2. Awareness: Raise awareness about communities’ acceptance of men’s use of power over women, which fuels HIV/AIDS and violence against women.
3. Support: Support women and men directly affected by or involved in these issues to change.
4. Action: Take action to prevent violence against women and HIV/AIDS.

Through each of these stages, the SASA! materials provide the framework for the delivery of mutually reinforcing messages delivered through both formal and informal sources. Thus, community members are repeatedly exposed, either directly or indirectly (for example through other community members who have attended activities) as the intervention gains momentum. Through this process, new ideas, attitudes and norms that promote more equitable relationships diffuse throughout the community and behaviours and community responses to violence are expected to shift.

In intervention communities, four types of activists delivered SASA! programming; (i) community activists (CAs), (ii) Ssengas (traditional marriage counsellors), (iii) drama groups and (iv) community based organisations. CAs and Ssengas received bi-monthly training and support from CEDOVIP staff members to plan, host or implement activities including public events such as community dramas and poster discussions, small group activities, and one-on-one ‘quick chats’ [9]. Drama group members and community based organisations also participated in regular training over the course of the intervention and contributed to the dissemination of SASA! materials and messaging through drama and participation in community events. Regular training was also provided to community stakeholders including health care workers, police and institutional leaders who incorporated the knowledge and skills gained through SASA! into their everyday work responding to violence, preventing violence, working to improve service delivery and influencing policy [9]. CEDOVIP staff members delivered all training sessions, supported activists in planning activities, acted as mentors and conducted field visits to assess and assist in the delivery of community based activities. Staff also organised public screenings of SASA! films. Raising Voices staff led the monitoring and evaluation activities including daily field visits, data entry, analysis and reporting. CEDOVIP management oversaw the overall implementation of the intervention with support from Raising Voices on a regular basis.

The SASA! approach is unique in that all activists and stakeholders are involved on a volunteer basis, receiving no stipends or payments in-kind in exchange for their involvement. This contributes to the development of a core of individuals in each intervention community with the skills necessary to continue disseminating SASA! messaging beyond the life of the intervention without the risk that once payments stop, the messaging will as well.

### The SASA! study

The SASA! study is a cluster randomised controlled trial conducted in eight communities in two administrative divisions in Kampala, Uganda. Sites were pair matched, with one from each pair randomly allocated to the intervention group and the other designated a control. Cross-sectional surveys of community members (men and women aged 18–49 years) were conducted at baseline (2007) and endline (2012) in both intervention and control sites. Over this four year time period programming was interrupted several times due to political disturbances, meaning that outcomes relate to approximately 2.8 years of intervention implementation [11]. Primary outcomes included measures of the social acceptance of gender inequality and intimate partner violence, women’s past year experience of intimate partner violence, community responses to women experiencing violence and sexual risk behaviour [11]. A cluster-level analysis compared outcome prevalence in intervention versus control communities at follow-up. The intervention showed positive impacts across all outcomes measured, as demonstrated by the adjusted risk differences presented in Table 1. For the purposes of this analysis we have used a 90 % confidence interval to characterise uncertainty around outcomes. This narrower confidence interval was used in place of the 95 % confidence intervals presented in the main trial analysis where the study was not powered to detect statistically significant changes in these outcomes. This was caused by a combination of having few clusters in the trial along with an unexpected increase in between-cluster variation in prevalence of the IPV outcomes between baseline and follow-up. Details of the study protocol [6], a full description of survey methodology and main trial results [11] are available elsewhere.

**Table 1:** SASA! cluster randomised controlled trial outcomes

	Intervention	Control	Unadjusted risk difference^a^(90 % CI)	Adjusted risk difference^b^ (90 % CI)
Reduced social acceptance of gender inequality and IPV				
Acceptability of physical violence by a man against his partner				
• Male attitudes	136/768 (18 %)^±^	544/634 (86 %)^±^	−68.2 (−88.0–-48.3)	−68.6 (−87.0–-50.3)
• Female attitudes	191/599 (32 %)^±^	311/528 (59 %)^±^	−27.5 (−42.6–-12.4)	−27.7 (−42.7–-12.6)
Acceptability that a woman can refuse sex				
• Male attitudes	744/768 (97 %)^±^	474/634 (75 %)^±^	22.3 (6.4–38.2)	21.9 (7.9–36.0)
• Female attitudes	542/599 (90 %)^±^	385/529 (73 %)^±^	18.1 (8.5–27.7)	19.0 (10.2–27.8)
Decrease in women’s experience of IPV				
Past year physical IPV	46/504 (9 %)	93/424 (22 %)	−12.8 (−24.6–-0.9)	−11.6 (−22.3–-0.9)
Past year sexual IPV	70/504 (14 %)	84/423 (20 %)	−5.9 (−15.8–4.0)	−6.0 (−15.9–3.9)
Improved response to women experiencing IPV				
Appropriate community response to women experiencing IPV in past year	28/102 (27 %)	18/139 (13 %)	14.0 (−6.0–33.9)	15.2 (−4.7–35.1)
Decrease in sexual risk behaviours				
Past year concurrent sexual partners among non-polygamous men partnered in past year	139/508 (27 %)	177/397 (45 %)	−17.3 (−28.1–-6.5)	−19.9 (−28.7–-11.0)

^a^Risk differences calculated at the cluster-level, all (crude and adjusted) adjusted for community-pair, and weighted according to the number of observations per village

^b^Adjusted risk differences generated on the basis of expected number of events from a logistic regression model on individual data with independent variables including age, marital status and EA-level summary baseline measure of outcome indicator

^±^Baseline measure controlled for: disclosed past year IPV and got helpful response

### Measuring costs and resource use

We estimated total costs related to (i) the development of the SASA! Activist Kit and (ii) implementation of the SASA! intervention in Kampala, Uganda. The time period considered for development of the SASA! Activist Kit was 2005–2008. Costing of SASA! implementation corresponds to approximately 4 years of programming from 2008–2012 and includes some start up activities conducted in 2007.

A provider perspective was adopted and both financial and economic costs considered. Resource utilisation data, including number of SASA! activities and activity inputs (equipment, supplies and personnel time) were collected retrospectively using both financial records and routine monitoring and evaluation data. Using complete financial records, a top-down costing approach was used to allocated financial costs associated with development of the SASA! Activist Kit, the SASA! Intervention and administrative and overhead costs. A bottom-up approach was used to quantify Activist time associated with implementation of community activities. Costs to individuals related to attending SASA! activities were not considered.

All costs were considered in the year in which they were incurred. Costs incurred in Ugandan Shillings were converted to US dollars using the average exchange rate [12] in the year in which expenditure occurred and converted annually to US dollars. Final costs were adjusted to 2011 US dollars using the World Bank GDP deflator [13]. Capital costs were annualised over the expected useful life for each item and a 3 % discount rate was applied throughout.

An incremental approach was used to assess the cost of developing SASA! materials over the course of three years from 2005 and 2008. Recurrent costs included: personnel costs, building and overhead, activities, supplies, and other. Other recurrent costs include technical support provided by Raising Voices to CEDOVIP, translation, artwork, film production, printing, shipping, field testing of materials and piloting activities. Capital costs included: building costs, equipment and materials development. Personnel costs were estimated based on a proportion of annual gross salary for Raising Voices and CEDOVIP staff or stipend for volunteer activists. Capital costs were valued using purchase prices obtained from financial records or replacement value estimated based on purchase price of similar items in the same financial year and annualised over their expected useful life using a discount rate of 3 % with the exception of building cost which were valued using annual rental rates. All costs were allocated toward SASA! materials development based on estimated proportion of use. Overhead costs were allocated using a simple step-down methodology whereby the cost of support services were allocated to final cost centres based on programme areas.

Overall, the cost of developing SASA! was treated as an initial investment expected to yield benefits beyond both the time periods in which costs were incurred and the specific intervention setting. As such, the total cost associated with materials development was treated as a single capital item. Annual total costs were annualised year on year over the course of development and the final total costs were annualised over 10 years using a 3 % discount rate. The final total was divided across the estimated number of roll out sites using SASA! materials annually between 2008 and 2011 (50 sites in 2008, 60 sites in 2009, 70 sites in 2010 and 80 sites 2011). The number of roll-out sites per year was estimated based on interviews on with Raising Voices staff responsible for distributing materials and providing technical support to organisations using the materials.

Implementation costs were estimated using a full economic costing approach covering the period 2007 to 2011. As with the cost of developing SASA! materials, cost categories included the same capital and recurrent costs with the addition of transport and training costs. Similarly, all costs were allocated based on proportion of use related to SASA! implementation, overhead and support costs allocated using a step-down approach and capital items were annualised over their expected useful life using a discount rate of 3 %.

As has been previously described, the SASA! intervention model relies heavily on activists to implement programming in the community on a volunteer basis. Activists also attended bi-monthly training sessions over the course of the intervention. The opportunity cost of this labour was valued using an estimated local hourly wage for temporary unskilled labour of 1500 Ugandan Shillings (2011 US$0.60) reflecting the reality that many activists were either casual labourers or not otherwise employed [14]. This is consistent with valuing volunteer time based on the market value of labour used in other community based interventions [15–18]. The total value of this contribution was calculated using an ingredients-based approach whereby the estimated time per activity was multiplied by the number of activities and the estimated hourly wage. Community activists and local leaders were supported in conducting SASA! activities by paid staff members whose time was allocated toward intervention activities based on individual interviews and confirmed by both Raising Voices and CEDOVIP management.

All research costs were excluded, however; routine monitoring and evaluation costs were included as it is expected that these costs would also be incurred in replication and scale up.

### Outcomes

Unit cost estimates for the economic analysis include the cost per activist supported (excluding community based organisations), cost per community member in intervention areas, cost per SASA! activity conducted. Cost effectiveness was estimated as the cost per case of physical IPV averted (year free of physical IPV).

The number of activists supported and number of activities conducted were obtained from routine monitoring and evaluation data. Monitoring data were compiled based on a combination of monthly activity planners submitted by each CA, reports of completed activities submitted by activists and quality assessment reports completed by CEDOVIP and Raising Voices staff based on observed field activities.

Monitoring data showing the number of SASA! activities conducted by activists was available from June 2009 to December 2011 based on individual reports of activities completed. The total number of activities conducted between January 2008 and May 2009 were estimated based on available data by calculating the average number of activities for the corresponding month in subsequent years and assuming that the number of activities were 40 % lower in 2008 and 20 % lower in 2009 in order to reflect an upward trend of programming over the intervention period. A similar approach was used to estimate the total number of activities conducted by Ssengas, drama groups and community based organisations where data were only available from 2010–2012. The incomplete monitoring data in the earlier part of the intervention reflects the challenges of implementing a complex community based intervention with reliant on individual activist reports. In the latter part of the intervention, a focus on routine data collection and monitoring protocols lead to substantial improvements in reporting. Reports may be biased by either over or under reporting; however, data are consistent with planned activities and field monitoring visits, making significant over reporting unlikely. Therefore, estimates presented in the base case are more likely to be an underestimate. Best and worst case scenarios are presented in sensitivity analysis.

The intervention’s ‘target population’ is considered the best proxy for number of people reached by SASA!, as the model of social diffusion on which the intervention is built means that community members may be exposed through indirect routes as well as through direct exposure to SASA! materials and activities. Indeed intervention impacts on trial outcomes were seen at the community level (not just among those directly exposed to SASA! materials or activities).

The target population for prevention of past year experience of IPV was considered to be women aged 18–49 years who had been in a regular or casual partnership in the past year, and who lived in a census enumeration area (EA) in which a SASA! community activist was operating. It should be noted that this is a conservative interpretation of the intervention’s target population - in reality, people outside this age bracket and beyond the boundaries of these enumeration areas will have been reached. However, since the trial examined intervention effects within this circumscribed population, this is the target population deemed appropriate to use for unit cost and cost-effectiveness estimates.

To estimate the target population size, we used data from both the SASA! follow-up survey and the SASA! household mapping exercise in which we listed every household in each EA in which a community activist was operating [10, 11]. The number of 18–49 year old women in the target population was estimated as the number of 18–49 year old women in the sampled households (data collected in the follow-up survey) divided by the sampling fraction (proportion of total mapped households completing the survey). Follow-up survey data was then used to estimate the proportion of these women who would have been partnered in the past year.

The measure of past year experience of physical IPV was based on a series of questions on whether the woman had experienced different physical acts by a partner – those who reported having experienced one or more of these acts in the past 12 months were coded as having past year experience of physical IPV (as described in the main results paper [11]). The number of cases averted was estimated as the difference between the number of cases that we would have expected to occur in the intervention communities in the absence of the intervention, and the number of cases actually observed in intervention communities. Observed cases were extrapolated by applying study estimates of IPV prevalence in the intervention communities, to the target population size. The adjusted risk difference in prevalence of past year IPV between intervention and control communities at follow-up was then used to estimate the additional number of cases that would have been observed in intervention communities had SASA! not been implemented (expected number).

Number of cases averted is then represented as follows:

$$ Cases\  averted = \left(R\_IP{V}_o-R\_P{V}_e\right)* Pop $$

Where *R_IPV*_*e*_ is the expected risk, *R_IPV*_*o*_ is the observed risk and *Pop* is the number of people in the target population.

The adjusted risk difference between intervention and control communities for IPV was calculated using a cluster-level analysis controlling for site pair, age and marital status, akin to the analysis of relative risk described in the main trial paper [11]. Ninety percent confidence intervals around the estimates of risk difference were used to calculate upper and lower bounds for the number of cases of IPV averted.

### Cost effectiveness

The cost effectiveness of the SASA! intervention was assessed compared to a ‘do nothing’ alternative, which in this case is represented by the costs and outcomes associated with the control groups in the intervention. The cost effectiveness ratio (CER) was calculated as follows:

$$ \mathrm{C}\mathrm{E}\mathrm{R}\ \mathrm{per}\ \mathrm{past}\ \mathrm{year}\ \mathrm{case}\ \mathrm{of}\ \mathrm{I}\mathrm{P}\mathrm{V}\ \mathrm{averted} = \kern0.5em \frac{TC}{Cases\  averted\ } $$

Using this approach the total cost of implementing the SASA! Intervention, represented by *TC*, is divided by the intervention effectiveness in terms of the estimated number of cases of violence averted in the target population.

### Sensitivity analysis

A number of the parameters for both the costing and the estimation of outcomes were characterised by uncertainty. To quantify the impact of uncertainty around assumptions related to costing methodology and inputs on unit costs, univariate sensitivity analysis was performed by varying the following key parameters:

- Number of SASA! activities conducted +/−50 %
- Discount rate varied to 0 % and 10 %
- Expected useful life of SASA! materials varied to 4 years (the length of the SASA! intervention) and 20 years
- Value of community activist time doubled (2011 US$1.20/hour) and set to zero
- Number of SASA! roll out sites that the cost of materials development are applied to +50 % or 1 (the full cost of materials development applied to the SASA! intervention)

Sensitivity analysis around the impact of uncertainty in outcome parameters on cost effectiveness was performed by varying estimates of the number of cases averted (using the 90 % confidence interval around the intervention-control risk difference to determine the upper and lower bounds).

## Results

### Development of the SASA! activist Kit

The total estimated cost of developing the SASA! Activist Kit is 2011 US$138,598 (see Table 2). Recurrent personnel made up 76 % of the total costs (2011 US$105,322). Recurrent building and overhead costs, transportation, activity costs, supplies, training and other recurrent costs made up 21 % of the total costs (2011 US$29,165). Capital costs related to materials development comprised approximately 3 % of the total cost (2011 US$4052).

**Table 2:** Total cost of developing the SASA! Activist Kit, 2011 US$

	Total	Proportion of total
Recurrent Staff	105322	76 %
Recurrent Activist Opportunity Cost	0	0 %
Recurrent Building and Overhead	314	0 %
Recurrent Transport	0	0 %
Recurrent Activities	0	0 %
Recurrent Supplies	50	0 %
Recurrent Training	0	0 %
Recurrent Other	27638	20 %
Capital Building	1025	1 %
Capital Equipment	188	0 %
Capital Transport	0	0 %
Capital Training	9	0 %
Capital Materials Development	4052	3 %
Total	138598	100 %

### Intervention costs and cost effectiveness

The annual and total costs of SASA! implementation are presented in Table 3. The estimated total cost of delivering the intervention is approximately 2011 US$553,252. Of this total, approximately 97 % are recurrent costs and 3 % are capital costs. Personnel costs comprise 42 % of recurrent costs with the estimated economic cost of community activists’ time contributing approximately 3 % to the total.

**Table 3:** Annual and total cost of SASA! Implementation, 2011 US$

	2007	2008	2009	2010	2011	Total	Proportion of total
Recurrent Staff	0	38972	47466	58536	88736	233710	42 %
Recurrent Activist Opportunity Cost	0	3731	3982	5022	3488	16223	3 %
Recurrent Building and Overhead	0	1635	1778	2730	2651	8794	2 %
Recurrent Transport	0	4478	6491	10772	4927	26668	5 %
Recurrent Activities	0	11191	8619	33825	25708	79343	14 %
Recurrent Supplies	0	9618	4870	10193	4469	29150	5 %
Recurrent Training	0	18870	19275	39391	49318	126853	23 %
Recurrent Other	858	2990	1998	7367	507	12862	2 %
Capital Building	0	2204	2622	3058	1365	9249	2 %
Capital Equipment	15	761	291	1170	540	2763	0 %
Capital Transport	0	157	28	5	40	230	0 %
Capital Training	0	231	239	201	231	902	0 %
Capital Materials Development	0	281	4838	298	216	5632	1 %
Total	872	95120	102497	172567	182195	553252	100 %

Over the course of the intervention 351 community activists and local leaders worked to deliver approximately 11,877 SASA! activities. The estimated total cost of supporting activists to deliver programming over the course of the intervention is 2011 US$1576, or 2011 US$394 annually. The estimated cost per activity conducted is 2011 US$47 per activity (see Table 4).

**Table 4:** Unit cost and cost effectiveness estimates of SASA! implementation, 2011 US$

Indicator	Average Cost (2011 US$)
Unit cost	
Per person in intervention community (10 year+)	16
Per person in intervention community (15 years+)	18
Per person in intervention community (18–49 years)	21
Per activist supported	1576
Per activist supported per year	394
Per activity (base case)	47
Cost effectiveness	
Per case of past year physical IPV averted	460

The total target population (women aged 18–49 years who had been in a regular or casual partnership in the past year and who lived in a census enumeration area in which a SASA! community activist was operating) was estimated as 10,333. The total cost per person in the target population is therefore estimated to be approximately 2011 US$21, or 2011 US$5 annually. Expanding this to individuals 15 years of age or more or aged 10 and above, the estimated unit cost is 2011 US$16–18 (see Table 4).

Approximately 1202 cases of past year physical IPV were averted (90 % CI: 97–2307). This translates to an average cost of 2011 US$460 per case of past year IPV averted.

### Sensitivity analysis

The results of univariate sensitivity analysis showed that unit cost estimates are robust to variations in key parameters (see Table 5). With the exception of the cost per activity, the lowest unit cost estimates are associated with setting the value of volunteer time equal to the financial cost (which is zero), and the highest unit cost estimates are associated with allocating the cost of SASA! materials entirely to the intervention sites.

**Table 5:** Univariate sensitivity analysis for unit cost and cost effectiveness estimates for SASA! implementation, 2011 US$

	Low (2011 US$)	Percent change from base	High (2011 US$)	Percent change from base
Unit cost				
Per person in intervention community (10 year+)	18	−2	20	12
Per person in intervention community (15 years+)	15	−3	17	10
Per person in intervention community (18–49 years)	21	−2	24	12
Per activist supported	1530	−2	1761	12
Per activist supported per year	382	−2	440	12
Per activity	31	−48	93	51
Cost effectiveness				
Per case of past year physical IPV averted	447	−2	514	10

**Fig. 1:**
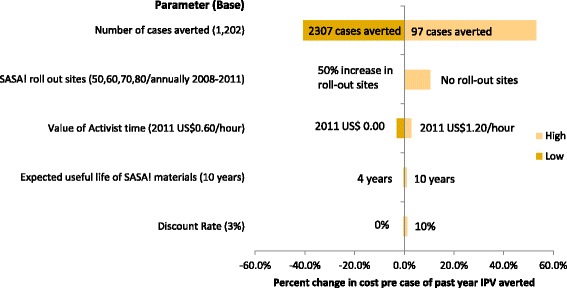
Tornado diagram showing sensitivity of cost per case of past year physical intimate partner violence averted to key assumptions

Sensitivity analysis for the cost effectiveness analysis was extended to consider the impact of uncertainty in the measure of intervention effectiveness; these results are presented in Fig. 1. Overall, the results are not sensitive to changes in assumptions around cost and input parameters, but are highly sensitive to uncertainty around the outcome estimate. Using the upper and lower bounds of the 90 % confidence interval around the estimate of the number of cases of IPV averted the cost per case of past year violence averted ranges from 2011 US$327–982.

## Discussion

The total estimated economic cost of delivering the intervention was 2011 US$553,252, or approximately 2011 US$5 per person in the intervention communities per year. This compares favourably to other violence prevention interventions and gender responsive HIV interventions and empowerment interventions [7]. However, evidence regarding the cost and cost effectiveness of interventions aimed at preventing IPV in low income settings is scarce. Assessing the relative value for money associated with such interventions therefore remains a challenge.

The only other published study estimating the unit costs of preventing violence in sub-Saharan Africa is related to the Intervention with Microfinance for AIDS and Gender Equity (IMAGE) study in South Africa [9]. Here the cost per person reached in the trial phase was 2011 US$49.17 (2004 US$42.93) and 2011 US$14.75 (2004 US$12.88) per person when the programme was scaled up. The latter estimate is comparable to the cost per person in the SASA! communities in the trial phase which ranged from 2011 US$15 – US$23 depending on the target population considered. In terms of cost effectiveness, our estimate of the cost per case of past year of violence averted in SASA! (2011 US$485) is considerably less than that the estimate of 2011 US$813 per year free of IPV reported in the IMAGE trial phase [19]. In the scale up phase, the cost per year free of IPV reported in IMAGE fell to 2011 US$244, approximately 30 % of the trial phase estimate. Expansion of SASA! activities may lead to similar economies of scale.

Importantly, the costing of the IMAGE study utilised an incremental approach, which considers only the cost of adding an intervention onto existing services, whereas the present analysis utilised a full economic costing approach which includes all administrative and overhead costs associated with implementing the intervention. Were an incremental costing used for the SASA! study, indirect overhead and administrative costs would not have been included and both unit cost and cost effectiveness estimates would be even lower. However, an incremental costing approach implicitly assumes that organisations have the administrative and managerial capacity to add additional programmes at little or no cost and this approach was not considered appropriate in the present case given that SASA! represented the bulk of programme activities for the implementing organisation. Further, for organisations in low and middle income countries considering implementing SASA! the assumption that the programme can be added onto existing infrastructure at no cost may not be appropriate, making full economic costs more useful for budgeting and planning purposes.

A recent systematic review of the costs and cost effectiveness of gender responsive interventions for HIV identified several economic evaluations of interventions focusing on gender empowerment and mitigating the impacts of violence [7]. Unit cost estimates for gender empowerment programming ranged from 2011 US$18.70 per participant reached in a community mobilisation and gender empowerment intervention for sex workers in India to 2011 US$158 per participant in a peer education programme in Brazil [7]. Unit cost estimates for the provision of post rape care ranged from 2011 US$30.10 per survivor in Kenya (without post-exposure prophylaxis for HIV) to 2011 US$819 per survivor in a study modelling the national scale up of post rape care services in South Africa. Again, unit cost estimates associated with the SASA! intervention compare favourably.

Sensitivity analysis showed that unit cost estimates are generally robust to key assumptions in the cost analysis. However, estimates for the cost per activity are sensitive to estimates of the number of activities conducted (which is reflected in the proportionate increase in costs). The variation in this parameter was selected in order to show best and worst case scenarios rather than likelihood of over reporting and the actual cost per activity is likely to tend toward the lower end. Further, this parameter does not impact outcome measures and so does not influence the overall cost effectiveness ratio. Complete monitoring data would reduce the uncertainty around this estimate. In future work this may be facilitated by a prospective study design.

Sensitivity analysis around key assumptions in the cost effectiveness analysis also showed that the estimate of cost per case of past year physical IPV averted is sensitive to the number of cases averted, with the range of estimates reflecting uncertainty in this outcome parameter. However, varying the value of other parameter estimates had a minimal impact on the cost effectiveness ratio. In particular, varying assumptions around the value community activists, resulted in a 3 % change in the cost per past year case of physical violence averted (range: 2011 US$447–474).

The cost effectiveness analysis uses the number of cases of physical IPV averted as estimated from trial data as the primary outcome. These figures should be interpreted as conservative estimates. In reality, impacts will have been felt among women outside the 18–49 year age bracket, and beyond the boundaries of enumeration areas in which CAs were operating. However, since effect sizes were measured exclusively among a population with these age and neighbourhood restrictions, we did not consider it appropriate to apply them more widely. It is also worth noting, that we have focused on cases of violence averted in the year preceding the endline survey (the fourth and final year of SASA! programming). However, it is reasonable to assume that additional cases of violence will have been averted in years two and three of the intervention, and that future cases of violence may also be prevented as a long term impact of the intervention. We have also focused on cases of violence completely averted, meaning that no acts of violence were experienced by a respondent. This approach does not account for the fact that some women in intervention communities may have experienced a reduction in frequency or severity of IPV without a total cessation of the violence.

Whilst only one outcome, prevention of physical IPV, has been used to calculate cost-effectiveness, it is clear that SASA! had broad ranging effects on multiple outcomes, including changing social norms and reducing sexual concurrency among men. These broad impacts have a bearing not only on the cost effectiveness of the intervention in relation to violence prevention programming, but also for the potential value for money such an intervention could offer if co-financed by several sectors (for example violence, HIV and reproductive health), a funding solution recently proposed for programmes with cross-sectoral benefits [7].

Given the retrospective costing design it was not possible to link costs to individual levels of exposure. Given a study design with more clusters it may have been possible to link cluster level costs and outcomes; however, the small number of clusters involved in the study would have limited the extent to which we could interpret variation in cluster-level costs or draw meaningful conclusions about correlations between cluster-level costs and outcomes. The lack of individual level cost and outcome estimates meant that it was not possible to conduct a more robust probabilistic sensitivity analysis to estimate the impact of parameter uncertainty on the likelihood that the intervention is cost effective. Instead we have used the upper and lower bounds of the 90 % confidence interval around the number of cases of IPV averted to show how the cost per case averted may vary. A prospective costing study design may have been useful in identifying individual level costs associated with exposure to the SASA! intervention; however, trial results showed that intervention effects were seen in individuals who were not directly exposed to SASA!, so a focus on the costs associated with direct exposure would have over-estimated the cost per person reached.

This study has some limitations. Firstly, results of the sensitivity analysis show that the estimate of cost effectiveness is highly sensitive to uncertainty in the outcome measure. This is not a surprising result given that the cost effectiveness ratio is calculated as a unit cost. Secondly, the specific outcome selected for the cost effectiveness study does not facilitate comparisons of value for money. The use of a composite final outcome measure such as a disability adjusted life year (DALY) would have enabled some comparison of the cost effectiveness of SASA! in relation to other programmatic approaches and other types of investments in health. While a DALY measure of the burden of disease associated with exposure to violence is currently available, this metric considers lifetime exposure to violence while the present study focused on averting experiences of violence in the past year. The current DALY estimate would provide a useful outcome measure for a study aiming to prevent lifetime experience of violence; however, in order to adequately assess the impact of preventing further violence among women who have already experienced it, a new DALY measure for past year exposure is required. Further research in this field would allow for more accurate comparisons of effectiveness across interventions aimed at preventing IPV as well as facilitating the comparison of value for money between these interventions and those aimed at improving other aspects of health and well-being.

## Conclusion

The results of the SASA! study, a community cluster randomised trial of a community mobilisation intervention aimed at preventing intimate partner violence suggest positive community level effects in terms of reducing IPV and changing harmful gender norms. This study provides estimates of the cost and cost effectiveness of delivering the SASA! intervention in Kampala, Uganda. The results of this analysis add to a small evidence base around the economic evaluation of preventing violence in low income settings and shows that both the costs and cost effectiveness of SASA! compare favourably to other approaches to gender empowerment and mitigation of the impacts of violence. Policy makers should therefore consider implementing SASA! more broadly.

## Abbreviations

DALY: disability adjusted life year
HIV: human immunodeficiency virus
IMAGE: intervention with microfinance for AIDS and gender equity
IPV: intimate partner violence
